# Pregnancy-related anxiety and its associated factors during COVID-19 pandemic in Iranian pregnant women: a web-based cross-sectional study

**DOI:** 10.1186/s12884-021-03694-9

**Published:** 2021-03-15

**Authors:** Zeinab Hamzehgardeshi, Shabnam Omidvar, Arman Asadi Amoli, Mojgan Firouzbakht

**Affiliations:** 1 Department of Reproductive Health and Midwifery, Sexual and Reproductive Health Research Center, School of Nursing and Midwifery, Mazandaran University of Medical Sciences, Sari, Iran; 2 Social Determinants of Health Research Center, Health Research Institute, Babol University of Medical Sciences, Babol, Iran; 3 Commette Student Research, Babol University of Medical Sciences, Babol, Iran; 4 Department of Nursing- Midwifery, Comprehensive Health Research Center, Babol Branch, Isalamic Azad University, Babol, Iran

**Keywords:** Coronavirus disease 2019, Pandemic, Pregnancy-related anxiety

## Abstract

**Background:**

Pregnancy is a risk factor for coronavirus disease 2019 (COVID-19). Pregnant women suffer from varying levels of pregnancy-related anxiety (PRA) which can negatively affect pregnancy outcomes. The aim of this study was to assess PRA and its associated factors during the COVID-19 pandemic.

**Methods:**

This web-based cross-sectional study was conducted in 2020 on 318 pregnant women purposively recruited from primary healthcare centers in Sari and Amol, Iran. Data were collected using questionnaires (PRAQ, Edinburg, KAP of COVID-19, CDA-Q and Demographic questionnaire), which were provided to participants through the social media or were completed for them over telephone. Data were analyzed with the linear regression and the logistic regression analysis, at the significance level of 0.05 using the SPSS software (v. 21).

**Results:**

Around 21% of participants had PRA, 42.1% had depression, and 4.4% had COVID-19 anxiety. The significant predictors of PRA were number of pregnancies (*P* = 0.008), practice regarding COVID-19 (*P* < 0.001), COVID-19 anxiety (*P* < 0.001), depression (*P* < 0.001), and social support (*P* = 0.025) which explained 19% of the total variance. Depression and COVID-19 anxiety increased the odds of PRA by respectively four times and 13%, while good practice regarding COVID-19 decreased the odds by 62%.

**Conclusion:**

Around 21% of pregnant women suffer from PRA during the COVID-19 pandemic and the significant predictors of PRA during the pandemic include number of pregnancies, practice regarding COVID-19, COVID-19 anxiety, depression, and social support. These findings can be used to develop appropriate strategies for the management of mental health problems during pregnancy in the COVID-19 pandemic.

## Background

Pregnancy is among the most important events in women’s life. It is associated with many different physical, emotional, and social changes [1]. Besides, pregnant women are concerned with fetal growth and their future responsibilities and hence, are prone to varying levels of psychological problems such as mood changes, fatigue, emotional disorders, mixed anxiety-depressive disorder, and pregnancy-related anxiety (PRA) [2]. PRA is a common problem among women during and after pregnancy [3].

PRA is defined as concerns, preoccupations, and fears related to pregnancy, delivery, neonatal health, and childrearing [4]. Anxiety is a normal response in stressful conditions [5]. Accordingly, healthy levels of PRA help pregnant women adhere to health-related recommendations [6]. However, severe PRA can negatively affect women’s physical and mental health and their children’s cognitive, emotional, and behavioral development [2]. Different studies reported the relationship of PRA with health-related problems such as intrauterine growth restriction [7], premature birth [8], cleft lip and palate, still birth, neonatal death [9], autism, hyperactivity, and neurodevelopmental disorders [10]. The prevalence of PRA in developing countries is 10–25% [11]. Studies in Iran assessed PRA using general anxiety questionnaires and reported prevalence rates of 32.5 and 40% [12, 13].

A major source of PRA is affliction by infectious diseases [14], particularly during epidemics and pandemics. Pandemics affect people and communities at different levels and cause disturbances in communities. They challenge psychological resilience and are usually associated with horror, stress, anxiety, sleep disorders, and negative attitudes towards health [5]. Previous studies reported that affliction by viral infections during the epidemics of H1N1 influenza, Middle East Respiratory syndrome (MERS), and Severe Acute Respiratory Syndrome (SARS) caused pregnant women negative clinical outcomes such as death, spontaneous abortion, premature birth, and fetal death [15, 16].

Coronavirus disease 2019 (COVID-19) was first observed in December 2019 in Wuhan, China, and rapidly spread throughout the world [17]. In January 2020, the World Health Organization introduced it as an international crisis [18]. In Iran, COVID-19 was first reported in February and then, it affected many people around the country. The Ministry of Health of Iran reported that by the middle of October 2020, the number of patients with COVID-19 and the number of its associated deaths in Iran were more than 935,000 and 47,000, respectively (http://ird.behdasht.gov.ir/2020.28.11). This study was conducted during the first peak of COVID - 19 outbreak, when little information was available about the disease and ways of spread of COVID − 19 in population, and there was no extensive studies about the disease. There was any definitive treatment or any vaccine against the COVID-19 [19].

The risk factors for COVID-19 include impaired immunity, history of respiratory or cardiac disorders, cancer, aging, obesity, and pregnancy [6, 20]. However, there are little information about effect of COVID-19 in pregnancy [21], Pregnancy-related physiologic changes in the anatomy and the function of the lungs together with impaired immunity put pregnant women at great risk for affliction by COVID-19 [17]. During epidemics and pandemics, factors such as fear over affliction and death and disturbances in daily activities due to the necessity of quarantining cause varying levels of anxiety [17]. Uncertainties over transmission routes and doubts about national readiness for pandemic management affect people’s adherence to preventive measures and cause them psychological strains [22, 23]. The sources of concern and anxiety for pregnant women during pandemics include concern over quarantine-related loneliness during and after delivery, limited access to healthcare services due to fear over affliction, increased requests for delivery through elective Cesarean section, concerns related to the frequent use of disinfectants, and concerns over child care, breastfeeding, and vaccination [24].

A recent study on 178 pregnant women in Italy during the COVID-19 pandemic reported that 46.6% of them had anxiety due to their fear over potential fetal anomalies caused by COVID-19, 65% of them had fear over intrauterine growth restriction, and 51% of them had fear over premature birth [5]. Studies during the MERS pandemic also showed that 80% of afflicted patients experienced fear [25, 26], isolation, social stigmatization, anxiety and anger during the two-week course of quarantine period [26].

To the best of our knowledge, limited studies have assessed PRA during the COVID-19 pandemic and no study has yet assessed it during the pandemic using PRA-specific questionnaires. Therefore, the present study was conducted to address this gap. The aim of the study was to assess PRA and its associated factors during the COVID-19 pandemic.

## Methods

### Design

This web-based cross-sectional descriptive-correlational study was conducted from April 17 to May 31, 2020.

### Participants

Study population comprised all pregnant women referred to primary healthcare centers in Amol and Sari, Iran. Inclusion criteria were having a healthy pregnancy without history of COVID - 19 disease, access to social media or telephone, no history of stressful life events in the past 6 months (including divorce, marriage, or significant loss), no affliction by mental health disorders, and no intake of psychiatric medications. The only exclusion criterion was voluntary withdrawal from the study. Sampling was performed purposively.

The pergnant women who were included in the study had a complete health file in primary health care centers. For sampling, we referred to primary healthcare centers in Amol and Sari, Iran, and created a list of eligible women and their telephone numbers. Then, midwives in the study setting called eligible women over telephone, invited them to the study, provided them with the study instruments over the WhatsApp application, and asked them to complete them. Study instruments for participants with no access to the WhatsApp application were completed through telephone-based interviews. In order to have confidential questionnaire, one of the researchers (AAA) filled them by information obtained through the telephone interviews.

Based on the rule of thumb of fifteen participants per item [27], as the PRAQ has seventeen items, sample size was calculated to be 300.

### Instruments

Study instruments were a demographic and midwifery characteristics questionnaire, the Edinburgh Postnatal Depression Scale, the Pregnancy Related Anxiety Questionnaire, the Corona Disease Anxiety Questionnaire, and a COVID-19 knowledge, attitude, and practice questionnaire. The questionnaire was in Persian.

The demographic and midwifery characteristics questionnaire included items on age, educational level, place of residence, occupation, level of family support, financial status, pregnancy wantedness, number of pregnancies, route of previous deliveries, and history of abortion, premature birth, bleeding during pregnancy, gestational diabetes mellitus, hypertension, and placental disorders.

The Edinburgh Postnatal Depression Scale is used for depression assessment during pregnancy and after delivery [28]. It contains ten multiple-choice questions on depression severity scored 0–3 (items 1, 2, and 4) or 3–0 (items 3 and 5–10). The total score of the scale is 0–30 and respondent with scores more than 12.5 are considered to have depression [29]. A former study reported the acceptable validity of this scale [30].

The Pregnancy Related Anxiety Questionnaire assesses pregnancy-related fears and concerns [31]. The short form of this questionnaire has seventeen items scored on a seven-point scale from 1 (“Definitely not true”) to 7 (“Definitely true”). The possible total score of the questionnaire is 17–119 with higher scores indicating greater anxiety. There is no cutoff point for the score of this questionnaire, while people with scores ranging from 65 to 70% total scores considered to have anxiety [32]. In the present study, scores greater than 66.3 were interpreted as anxiety. This questionnaire has acceptable validity and reliability [31].

The COVID-19 knowledge, attitude, and practice questionnaire was a researcher-made questionnaire developed based on the protocols recommended by the World Health Organization and the Ministry of Health of Iran for COVID-19 prevention and management. The knowledge part of this questionnaire had thirteen items on the transmission routes and the methods for the prevention and management of COVID-19.The knowledge items were about the signs and symptoms of the COVID − 19, prevention of the COVID − 19, transmission of the COVID- 19 virus from mother to child during pregnanc*y*, lactation during disease and treatment of the COVID − 19). Items were scored either zero (“No” or “I don’t know”) or 1 (“Yes), resulting in a possible total score of 0–13. Scores greater than 9 were interpreted as good knowledge about COVID-19. The attitude part of the questionnaire had two items on the controllability of the COVID-19 pandemic which were scored either zero (“Disagree” or “I don’t know”) or 1 (“Agree”). Therefore, the possible total score of the attitude part is 0–2 and score 2 shows good attitude. The practice part included three items on the use of gloves and facemask in outdoor areas and attendance at public places during the COVID-19 pandemic. Items were scored either zero (“Yes”) or 1 (“No”), resulting in a possible total score of 0–3 with score 3 showing good practice. The validity of this questionnaire was assessed through face and content validity assessments. Eleven experts rated the items, and the content validity indices (CVI) of the three parts of the questionnaire were calculated to be 0.81–1. The Cronbach’s alpha of the questionnaire was 0.79.

The Corona Disease Anxiety Questionnaire (CDA-Q) was developed in Iran with eighteen items on physical and mental anxiety. Items are scored on a four-point scale as follows: zero: “Never”; 1: “Sometimes”; 2: “Mostly”; and 3: “Always”. The possible total score of this question is 0–54 and respondents with scores greater than 37.8 are considered to have anxiety. This questionnaire has acceptable validity and reliability [33].

### Data analysis

Data were analyzed using the SPSS software (v. 21.0). The measures of descriptive statistics were used for data description, while the linear regression analyses were used to determine the predictors of PRA. The logistic regression analyses were used to odd ratio of PRA. Since there were many variables in the study (City, Health Center, Age, Education, Residence, job, Husband job, Social support, Gravid, Abortion, Pre-term labor, Pain, Vaginal delivery, any medical history, Knowledge, Attitude, Practice, PRAQ, CDA-Q, Depression), variables which had relationship with PRA at a significance level of less than 0.2 were entered in the regression models. Data were analyzed at the significance level of 0.05.

## Results

**Fig. 1:**
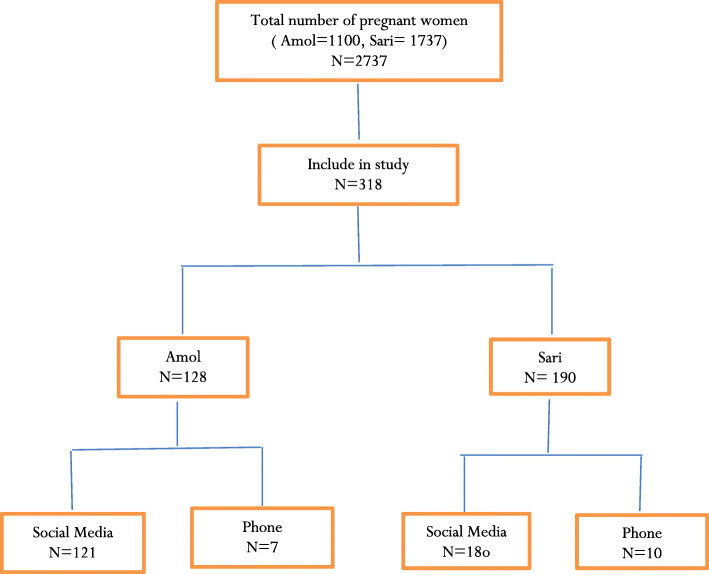
Flow chart of study

During the two-month course of data collection, the study instrument was viewed 942 times and 318 pregnant women completed them (response rate: 53%). The mean of response time was 16 min and 96% of participants completed the instruments via the WhatsApp application. The flow chart participants’ recruitment showed in the Fig. 1.

The mean of participants’ age was 28.57 ± 5.06 years and most of them had university degree (54.7%), were housewife (85.5%), lived in urban areas (67%), were primiparous (59.7%), and had good social support (50.6%) (Table 1).

**Table 1:** Participants’ demographic and midwifery characteristics

Characteristics	Mean ± SD or N (%)
Age (Years)	28.57 ± 5.06
Educational level	
Below diploma	32 (10.1)
Diploma	112(35.2)
University	174(54.7)
Occupation	
Employed	46(14.5)
Housewife	272(85.5)
Place of residence	
Rural areas	105(33)
Urban areas	213(67)
Husband’s occupation	
Health-related fields	20(6.3)
Other	268(84.3)
Unemployed	30(9.4)
Social support	
Good	161(50.6)
Moderate	142(44.7)
Poor	15(4.7)
Number of pregnancies	
1	190(59.7)
2	100(31.4)
3	174(54.7)
History of abortion	
Yes	75(23.6)
No	243(76.4)
History of premature delivery	
Yes	9(2.8)
No	309(97.2)
History of bleeding during pregnancy	
Yes	44(13.8)
No	274(86.2)
History of pain	
Yes	83(26.1)
No	235(73.9)
History of serious health problems	
Yes	32(10.4)
No	295(81.4)

The mean scores of participants’ knowledge, attitudes, and practice regarding the COVID-19 pandemic were respectively 9.17 ± 2.02 (in the possible range of 0–13), 0.95 ± 0.86 (in the possible range of 0–2), and 2.59 ± 0.63 (in the possible range of 0–3). Moreover, the mean scores of their PRA, depression, and COVID-19 anxiety were respectively 44.64 ± 19.93 (in the possible range of 17–119), 12.16 ± 3.43 (in the possible range of 6–24), and 10.58 ± 8.51 (in the possible range of 0–45) (Table 2). Most participants had limited knowledge (53.1%), poor attitude (65.1%), and poor practice (66.7%) respecting COVID-19. Moreover, 20.8% of them had PRA, 42.1% had depression, and 4.4% had COVID-19 anxiety (Table 2).

**Table 2:** The mean scores of the main study outcomes

Outcomes	Mean ± SD	Range	Level	N (%)
Knowledge regarding COVID-19	9.17 ± 2.02	0–13	Poor	169 (53.1)
			Good	149(46.9)
Attitude regarding COVID-19	0.86 ± 0.95	0–2	Poor	207(65.1)
			Good	111(34.9)
Practice regarding COVID-19	0.63 ± 2.59	0–3	Poor	106(33.3)
			Good	212(66.7)
PRA	19.93 ± 44.64	17–119	Yes	66(20.8)
			No	252(79.2)
Depression	3.43 ± 12.16	6–24	Yes	184(42.1)
			No	134(57.9)
COVID-19 anxiety	8.51 ± 10.58	0–45	Yes	14(4.4)
			No	304(95.6)

Linear regression analysis using the stepwise method showed that the significant predictors of PRA were number of pregnancies (*P* = 0.008), practice regarding COVID-19 (*P* < 0.001), COVID-19 anxiety (*P* < 0.001), depression (*P* < 0.001), and social support (*P* = 0.025). These variables explained 19% of the total variance of PRA mean score (Table 3).

**Table 3:** The results of linear regression analysis for PRA prediction

	Model1^a^						Model 2^b^					
	B	95% CI		*P*- value	R^2^	AR^2^	B	95% CI		*P* value	R^2^	AR^2^
		LL	UL					LL	UL			
Age	0.048	−0.232	0.613	0.376	41.6	38.2					19.8	18.7
Place of residence	0.077	−0.724	7.232	0.108								
Occupation	0.005	−5.091	5.682	0.913								
Number of pregnancies	−0.166	−8.568	−1.627	0.004			−0.120	−6.404	−0.980	0/008		
Social support	0.101	0.119	6.806	0.042			0.101	0.434	6.503	0.025		
Covid-19 anxiety	0.410	0.737	1.202	< 0.001			0.415	0.754	1.209	< 0.001		
Depression	0.228	0.752	1.898	< 0.001			0.218	0.707	1.814	< 0.001		
Knowledge regarding COVID-19	−0.004	−0/969	0.896	0.939								
Practice regarding COVID-19	−0.180	−8.54	−2.777	< 0.001			−0.177	−8.367	−2.790	< 0.001		
Attitude regarding COVID-19	−0.028	−2.733	1.456	0.549								

^a^Unadjusted Model

^b^Adjusted Model

The logistic regression analysis also showed that the number of pregnancies (one vs. more), history of serious health problems (diabetes mellitus, renal disease, and hypertension vs. others or none), knowledge (score above 9) and practice respecting COVID-19 (good vs. poor), and COVID-19 anxiety (yes vs. no) were eligible variables for logistic regression analysis. The results of the analysis showed that depression increased the odds of PRA by four times (adjusted odds ratio: 4.298, 95% confidence interval: 2.161–8.546, *P* < 0.001) and COVID-19 anxiety increased the odds of PRA by 13% (adjusted odds ratio: 1.125, 95% confidence interval: 1.066–1.187, *P* < 0.001). Good practice respecting COVID-19 also decreased the odds of PRA by 62% (adjusted odds ratio: 0.379, 95% confidence interval: 0.188–0.765, *P* < 0.001) (Table 4).

**Table 4:** The results of logistic regression analysis to determine the odds of PRA

		B	*P*- value	AOR	95% CI	
					LL	UL
Number of pregnancies	1	0.775 Ref.	0.246	2.170	0.586	8.042
	> 1					
Practice regarding COVID-19	(score above 2) Good	−0.969 Ref.	0.007	0.379	0.188	0.765
	(score under 2) Poor					
Knowledge about COVID-19	(score above 9) Good	−0.085 Ref.	0.482	0.919	0.725	1.164
	(score under 9) Poor					
COVID-19 anxiety	(score above 37.8) Yes	0.118 Ref	< 0.001	1.125	1.066	1.187
	(score under 37.8) No					
Depression	(score abov12.5) Yes	1.458	< 0.001	4.298	2.161	8.546
	(score under 12.5) No	Ref				
History of health problems	Yes (Diabetes mellitus, hypertension, renal disease)	1.286 Ref	0.055	3.617	0.971	13.480041
	No or others					
constant		−2.247	0.1	0.084		

## Discussion

This study aimed to assess PRA and its predictors during the COVID-19 pandemic. Findings showed that almost 21% of the participants experienced PRA. This is in line with the findings of a former study in Iran [34]. In a systematic review a prevalence of anxiety was reported from 3.8 to 17.5% in Asian countries [35] .A study in China also showed that the prevalence of moderate to severe PRA was 28.8% [36]. The COVID-19 pandemic has reduced pregnant women’s access to routine prenatal care services due to factors such as rapid spread of the disease, lack of an effective treatment or vaccine, the necessity of quarantining and its subsequent loneliness during affliction, stigmatization and despair [37], and concerns over contamination with the virus in healthcare settings [11]. Moreover, it has caused pregnant women different psychological disorders due to the restriction of the number of companions in healthcare settings, concerns over the lack of workforce in maternity settings, limited access to specialized care services for women with suspected COVID-19 [38], and requests for pregnancy termination or elective Cesarean section surgery [39].

Findings showed that pregnant women with COVID-19 anxiety were 13% more likely to have PRA. Moreover, those with diabetes mellitus, renal disease, or hypertension were 3.6 times more at risk for PRA. Contrarily, participants with good practice respecting COVID-19 were 62% less likely to have PRA. These findings imply that the COVID-19 pandemic is a serious challenge for pregnant women. A former study in Iran also showed that pregnant women had higher levels of COVID-19 anxiety compared with their non-pregnant counterparts [40]. Another study in Iran revealed that pregnant women in the third trimester of pregnancy were concerned with COVID-19 disease and its consequences and had higher levels of anxiety. That study also reported that higher levels of anxiety among pregnant women during the COVID-19 pandemic might be due to their limited access to healthcare services, their concerns over the unsafe environment of healthcare settings, and concerns over affliction by the disease [34]. A study in Pakistan showed that 84% of pregnant women had fear over COVID-19 and the mean score of generalized anxiety disorder among women with fear over COVID-19 was significantly higher [41].

Our findings also showed the number of pregnancies as a significant predictor of PRA. Similarly, a former study found gravidity as a significant factor contributing to PRA symptoms and reported that nulliparous women had higher levels of PRA compared with their multiparous counterparts [42]. Moreover, we found that most participants had poor knowledge, attitude, and practice respecting COVID-19. Attitude and practice respecting COVID-19 are correlated with the level of knowledge [10, 43, 44]. Moreover, our findings showed that 42% of participants had depression. In a systematic review study the prevalence of depression was reported from 5.2 to 40% [35]. COVID-19 prevention largely depends on social distancing and quarantining [45] which in turn can cause social isolation, sadness, worries, anger, irritability, despair [46] and increasing level of domestic violence [35]. Studies on the general population in China reported that almost one third of them had depressive symptoms [47] and women had higher levels of depression and anxiety [18]. A study also showed that depressive symptoms have increased during the COVID-19 pandemic [42]. Anxiety and depression during the perinatal period are associated with depression in later years of life [48]. Moreover, PRA can increase the risk of antenatal depression [49] and pregnancy-related complications such as preterm birth [50]. Contrary to our findings, a former study reported no significant difference in depression rate among pregnant women before and after the SARS outbreak [8]. A study in Iran also showed no significant difference among non-pregnant, pregnant, and breastfeeding women regarding depression rate during the COVID-19 pandemic [40].

### Strengths and limitations

One of the main strengths of the present study was web-based sampling and data collection which helped us collect the necessary data without exposing participants to COVID-19. However, web-based data collection can increase the risk of biases [51]. For instance, as people with better socioeconomic status may have better access to social media, web-based data collection may be associated with some levels of selection bias. We attempted to prevent and manage this bias through telephone-based data collection from participants with limited access to social media. There is a data collection limitation. Data collection through telephone may also associate with bias at data assessor level. As the total number of participants that completed the questionnaire via telephone were 17 person, it didn’t seem to affect the results of the study. This study was done in a cross-sectional mode that does not show any causal relationship between variables.

## Conclusion

This study shows that around 21% of pregnant women suffer from PRA during the COVID-19 pandemic and the significant predictors of PRA during the pandemic include number of pregnancies, practice regarding COVID-19, COVID-19 anxiety, depression, and social support. Although the effects of COVID-19 on pregnancy outcomes are still unknown, COVID-19 can negatively affect pregnancy outcomes through causing different mental health problems such as anxiety. Lack of definitive treatment of COVID-19, limited information on the effect of the disease on pregnant mother and neonate, uncertainty about how long the quarantine is to be in action, fear of attending hospital and health care centers were some reasons on anxiety during quarantine COVID-19.

### Implications

Healthcare authorities can use the findings of the present study to accurately identify pregnant women who are at higher risk for PRA and employ strategies to reduce their PRA through improving their knowledge, attitude, and practice regarding pandemics.

## Data Availability

The datasets analyzed during the current study are available from the corresponding author on reasonable request.

## Abbreviations

COVID-19: Coronavirus disease 2019
PRA: Pregnancy-related anxiety
MERS: Middle East Respiratory syndrome
SARS: Severe Acute Respiratory Syndrome
SPSS: Statistical Package for Social Sciences
SD: Standard Deviation
AOR: Adjusted Odd Ratio
CI: Confidence Interval
